# The first report of triple anthelmintic resistance on a French Thoroughbred stud farm

**DOI:** 10.1016/j.ijpddr.2024.100528

**Published:** 2024-02-23

**Authors:** Aurélie Merlin, Nicolas Larcher, José-Carlos Vallé-Casuso

**Affiliations:** a Anses, Laboratory for Animal Health in Normandy, Physiopathology and Epidemiology of Equine Diseases Unit, 14430 Goustranville, France; b Mixed Technological Unit “Equine Health and Welfare – Organisation and Traceability of the Equine Industry” (UMT SABOT), France

**Keywords:** Horses, Strongylids, Anthelmintic resistance, Faecal egg count reduction test

## Abstract

This study assessed the anthelmintic resistance in strongylid nematodes against commonly used anthelmintic (AH) drugs in a French galloping racehorse stud farm from March to December 2023. Faecal egg count reduction tests (FECRTs) were conducted in three different groups of Thoroughbred yearlings (a group of 6 males, a group of 13 females and a group of 8 females and 3 males) following the new World Association for the Advancement of Veterinary Parasitology (WAAVP) guidelines. The efficacy of fenbendazole was tested in two groups once during the monitoring period (in March), the efficacy of ivermectin in 3 groups twice (in March–April and in November–December) and the efficacy of pyrantel in one group once (in May–June). For each FECRT, the 90% confidence interval of the percentage faecal egg count reduction was calculated using the hybrid Frequentist/Bayesian analysis method. The resistance in strongylids was observed to fenbendazole, pyrantel and ivermectin in all the groups in which these drugs were tested. The number of animals in each group was sufficient to reach ≥80% power for the resistance test. The results highlight the first case of triple AH resistance in strongylids in France. Further studies involving more farms and equids are required to assess the prevalence of AH resistance in France and refine recommendations for owners.

## Highlights

• Efficacy of anthelmintics assessed by strongylid faecal egg count reduction tests.
• First confirmed case of resistance to ivermectin in a French stud farm.
• Resistance on the same farm to all three classes of licenced anthelmintics.
• Racehorse movements could facilitate the spread of anthelmintic-resistant worms.

## Graphical abstract

**Figure:**
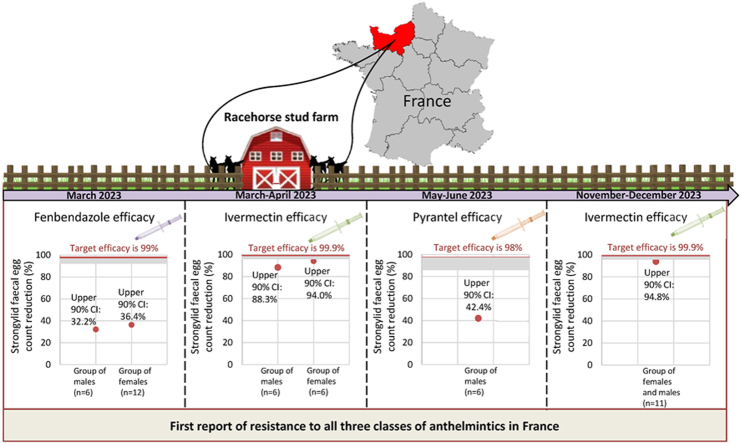

## 1 Introduction

Cyathostomins (Small Strongyles: Nematoda, Strongylida) are currently the most abundant gastrointestinal parasites of grazing equids regardless of the time of year (Collobert-Laugier et al., 2002; Corning, 2009). They are responsible for over 99% of the strongylid eggs shed in the faeces (Butler et al., 2021). Usually, cyathostomins are considered to be of low pathogenicity and most infected animals are not clinically affected. However, heavy burdens of adult cyathostomins accumulating in the large intestinal lumen may cause clinical symptoms such as ill-thrift, weight loss, colic, or diarrhea (Corning, 2009). In addition, cyathostomin larval stages can cause even more severe problems. The emergence of larvae from the cyst into the gut lumen, at the end of the winter/beginning of spring, may induce larval cyathostominosis resulting in sudden diarrhea, severe dehydration and serious colic with a mortality rate up to 50% in young animals (Love et al., 1999; Corning, 2009).

To avoid any negative impact on the health and growth of equids, the control of strongylids is usually performed with suppressive anthelmintic (AH) treatment several times throughout the year without assessing the specific risk. The three licenced AH classes in equids are i) benzimidazoles (fenbendazole-FBZ), ii) tetrahydropyrimidine (pyrantel-PYR) and iii) macrocyclic lactones-ML (ivermectin-IVM and moxidectin-MOX). Unfortunately, repeated, systematic treatments have inevitably led, over time, to the selection of resistant parasite populations. Resistance of equine strongylids to FBZ and PYR has been documented worldwide for several decades (Nielsen, 2022). Concerning the efficacy of ML, reduced egg reappearance periods or sporadic cases of resistance in equine strongylids have been reported from various parts of the world (i.e., Europe, UK, North and South America and Oceania) over the last 15 years (Nielsen, 2022).

Among groups of breeds, racehorses, mostly Thoroughbreds are particularly at risk of developing AH resistances due to frequent AH treatments with little or no parasite surveillance (Comer et al., 2006; Robert et al., 2015). In this equine population, the resistance of strongylids to ML (IVM and/or MOX) has already been reported in the USA (Nielsen et al., 2020), Australia (Abbas et al., 2021, 2024 and the UK (Bull et al., 2023).

In France, the few studies that have evaluated the effectiveness of AH against equine strongylids have been carried out 7–12 years ago in equestrian facilities including racehorses (Thoroughbreds and French trotters; Traversa et al., 2012; Geurden et al., 2013; Sallé et al., 2017). Those reported widespread resistance to FBZ and some evidence of resistance to PYR. In addition to that, the ML resistance was tested but not found.

Given the impact of strongylids on equine health, it is essential to monitor the effectiveness of AH in this group of breeds, in particular, to be able to adapt the recommendations on the use of AH and delay the emergence and spread of resistance.

This short communication presents a case of strongylids resistant to three classes of AH (FBZ, PYR and IVM) in Thoroughbred yearlings from the same French farm.

## 2 Materials and methods

### 2.1 Study population

This study was conducted in a galloping racehorse stud farm (Thoroughbreds and French chasers) in Normandy (France) between March and December 2023. Prior to the start of the study, the owner of the farm suspected resistance to FBZ and IVM because, in his opinion, the general condition and the growth of the animals remained unsatisfactory after the use of these AH classes. However, he had no concern about the effectiveness of PYR when the study started.

**Fig. 1:**
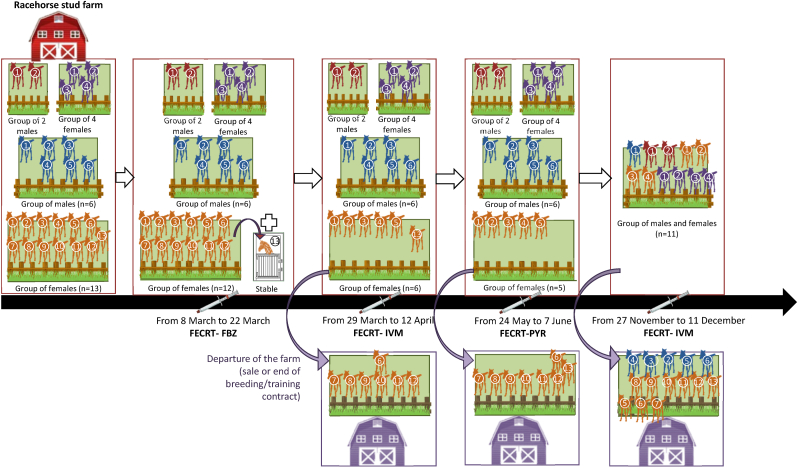
Implementation of faecal egg count reduction tests in Thoroughbred yearlings kept at a racehorse stud farm. FECRT, faecal egg count reduction test; FBZ, fenbendazole; IVM, ivermectin; PYR, pyrantel.

In this farm, the Thoroughbred male yearlings (n = 8) and the Thoroughbred female yearlings (n = 17) naturally infected by strongylids were enrolled in this study. Animals were born between January and June 2022 in France (in the farm recruited, n = 10 females + 5 males), Ireland (n = 6 females + 3 males) or England (n = 1 female). Animals born abroad were imported into France around the age of four months and they were treated with an AH treatment every one to two months from the age of one to two months. Animals born in France were treated with an AH treatment with the same frequency, but started being treated at four months of age. From birth and before participating in this study, they were given between three to six different AH treatments; mainly IVM but also PYR and FBZ according to date of birth. At the beginning of the study, all yearlings of the farm were divided into four groups: i) a group of 6 males, ii) a group of 2 males, ii) a group of 13 females and iii) a group of 4 females (Fig. 1). From March to September, each group grazed on different pastures (rotational grazing). During the season, the composition of the groups changed due to horses leaving the farm permanently for various reasons (the sale of animals or the end of a breeding contract). From September to December, the remaining yearlings have been grouped (n = 11) to graze on several pastures (rotational grazing).

### 2.2 Faecal egg count reduction tests (FECRTs)

The efficacy of the usual AH treatments planned by the veterinarian of the farm in yearlings from March to December 2023 was assessed using the available standardised method, the faecal egg count reduction test (FECRT), following the new World Association for the Advancement of Veterinary Parasitology (WAAVP) guidelines (Kaplan et al., 2023). The FECRT was carried out in groups of yearlings respecting the following conditions before each test: i) > five animals, ii) no deworming in the last two months, iii) grazing together for three months to ensure that they were infected by the same parasite community, and iv) no change to the group during the test (grazing together and no movement of equids outside the farm).

### 2.3 Anthelmintic classes tested

Each AH treatment was orally administered by the veterinarian of the farm: i) FBZ paste (Panacur®, Intervet, Angers Technopole, Beaucouze, France) at the recommended dose of 7.5 mg/kg body weight; ii) IVM paste (Eqvalan®, Boehringer Ingelheim Animal Health, Lyon, France) at the recommended dose of 200 μg/kg body weight, and iii) PYR paste (Strongid®, Zoetis, Malakoff, France) at the recommended dose of 6.6 mg/kg body weight. MOX was not used in yearlings. Appropriate individual AH dose was calculated from the weight of each animal, measured with a calibrated weight scale.

### 2.4 Faecal samples and analysis

To carry out each FECRT, faecal samples were collected from each animal of the group directly from the rectum by the farm owner on the day of the AH treatment, just before administration, and 14 days post-treatment.

After being kept at +4 °C for 24h, faecal samples were sent to the laboratory for Animal Health (Normandy site, Anses) by express courier with ice packs. At the laboratory, all faecal samples were stored under refrigeration (+4 °C) and examined within four days of collection to reduce the effect of egg degradation (Nielsen et al., 2010).

Strongylid faecal egg counts (FECs) were carried out using the modified McMaster technique (Raynaud, 1970) on 5 g of faeces diluted in 70 mL of sodium chloride solution with a relative density of 1.2 (sensitivity of 15 eggs per gram of faeces-EPG).

### 2.5 Required sample size to obtain a conclusive result

The required sample size to obtain ≥80% power for each statistical resistance test (FECRT-research protocol) was calculated via the web application available at https://www.fecrt.com, using i) the real arithmetic mean pre-treatment FEC, ii) the counting sensitivity of the coprocopic method used (15 EPG in our study), iii) the expected arithmetic mean efficacy for the anthelmintic used (in %) and iv) the non-inferiority margin (in %) (Denwood et al., 2023; Kaplan et al., 2023).

### 2.6 Interpretation of faecal egg count reduction test

FECRTs were only performed in groups that reached the minimum size needed to achieve a statistical power of at least 80% for the resistance test.

The individual/mean FEC data of pre- and post-treatment were analysed in the web application (https://www.fecrt.com). The 90% upper and lower confidence intervals (CI) of the percentage faecal egg count reduction at two weeks post-treatment were calculated using the Bayesian-Frequentist Hybrid Inference method (paired study design; research protocol; Denwood et al., 2023). Anthelmintic resistance to FBZ against strongylids was concluded if the upper 90% CI was less than an expected efficacy of 99% (lower efficacy threshold: 95%). Anthelmintic resistance to PYR was considered if the upper 90% CI was less than an expected efficacy of 98% (lower efficacy threshold: 88%). Anthelmintic resistance to IVM was defined if the upper 90% CI was less than an expected efficacy of 99.9% (lower efficacy threshold: 96%).

## 3 Results and discussion

During the monitoring period (March–December 2023), the yearlings received four different AH treatments (Fig. 1): i) a FBZ treatment in early March, ii) an IVM treatment at the end of March, iii) a PYR treatment at the end of May and iv) an IVM treatment at the end of November. In this farm, the animals also received an IVM treatment in January 2023. The frequency of AH treatment use per year was higher than that reported in a previous French study with managers/keepers of animals ≤2 years old susceptible to AH resistance (5 versus 1–3 AH treatments per year; Merlin et al., 2023).

Using the mean pre-treatment FECs obtained in each group of yearlings of more than five animals, the power calculation showed that these groups obtained the required number of horses to reach ≥80% power for the outcome: positive evidence of resistance should the efficacy be reduced (Table 1, Table 2).

**Table 1:** Efficacy of fenbendazole and pyrantel against strongylids in two groups of Thoroughbred yearlings.

Anthelmintic class tested	Period of FECRT (date of first FEC and treatment - date of second FEC)	Group	Participating horses (total number of horses in the group)	Total number of eggs counted before treatment	Mean (range) pre-treatment FEC in EPG	Mean (range) post-treatment FEC in EPG	Required sample size to achieve a statistical power of at least 80% for the resistance test	Number of animals shedding eggs before/after treatment	LCL and UCL at 90% CI at two weeks post-treatment	Test outcome
Fenbendazole	March 8 - March 22	Group of males	6 (6)	604	1510 (1035–2130)	1763 (465-2835)	N = 6	6/6	−100.9% - 32.2%	Resistant
	March 7- March 21	Group of females	12 (13)	703	879 (150-1815)	879 (90-1800)	N = 7	6/6	−57.3% - 36.4%	Resistant
Pyrantel	May 24 - June 7	Group of males	6 (6)	393	983 (165-2595)	228 (0–975)	N = 5	6/5	42.4%–96.6%	Resistant

CI, confident interval; EPG, eggs per gram of faeces; FEC, strongylid faecal egg count; FECRT, faecal egg count reduction test; LCL, lower confident interval; UCL, upper confident interval.

**Table 2:** Efficacy of ivermectin against strongylids in three groups of Thoroughbred yearlings.

Period of FECRT (date of first FEC and treatment - date of second FEC)	Group	Participating horses (total number of horses in the group)	Total number of eggs counted before treatment	Mean (range) pre-treatment FEC in EPG	Mean (range) post-treatment FEC in EPG	Required sample size to achieve a statistical power of at least 80% for the resistance test	Number of animals shedding eggs before/after treatment	UCL and LCL at 90% CI at two weeks post-treatment	Test outcome
March 29 - April 12	Group of males	6 males (6)	461	1153 (720-1785)	258 (45–555)	N = 5	6/6	57.2%–88.3%	Resistant
March 29 - April 12	Group of females	6 females (6)	571	1428 (225-2335)	178 (45–360)	N = 5	6/6	74.4%–94.0%	Resistant
November 27 - December 11	Group of females and males	8 females and 3 males (8 females and 3 males)	510	695 (105-1815)	74 (0–180)	N = 5	11/10	78.5%–94.8%	Resistant

CI, confident interval; EPG, eggs per gram of faeces; FEC, strongylid faecal egg count; FECRT, faecal egg count reduction test; LCL, lower confident interval; UCL, upper confident interval.

The efficacy of FBZ was first tested in a group of males (n = 6) and in a group of females (n = 12, the 13th female was excluded because it had to stay in the stable for a few days for an orthopaedic problem) (Fig. 1). Before FBZ treatment, all males could be classified as “high shedders” as they shedded >500 EPG according to the AAEP guideline (AAEP - American Association of Equine Practitioners, 2019). In the group of females, 58% of animals (7/12) could be considered as “high shedders” and the minimum FEC for “low shedders” was 150 EPG. Fourteen days post-treatment, all the horses in both groups continued to shed strongylid eggs. The FECs increased between pre- and post-treatment in 4/6 males and 8/12 females. According to the new WAAVP guidelines, the resistance to FBZ against strongylids was identified in both groups (Table 1). This result is consistent with previous studies from around the world, including France, which reported widespread and well-established resistance patterns in strongylids against this molecule (Nielsen, 2022). Indeed, confirmed resistance for FBZ was observed in 92–100% of the tested groups according to the previous French studies (Traversa et al., 2012; Geurden et al., 2013; Sallé et al., 2017).

As the treatment with FBZ did not reduce egg excretion by the animals (same high mean pre and post-treatment FEC; Table 1), an IVM-based treatment was administered 21 days after the previous FBZ treatment to avoid any impact on the health and growth of the yearlings (Fig. 1). As the interval between the two treatments was short, it is possible that the subpopulation of parasites within the animals may have undergone drug selection. Despite a possible persistence of FBZ activity on the worm population (which nevertheless seems minor given the results), the efficacy of IVM was tested in the same group of males and females. However, the size of the group of females was reduced (from 13 to 6 animals) due to the sale of animals or the end of breeding contract (Fig. 1).

Before IVM treatment, 100% of males (6/6) and 83% of females (5/6) could still be considered as “high shedders” (Table 2). The low shedder female exhibited a pre-treatment FEC of 220 EPG. Fourteen days post-treatment, all the animals in both groups continued to shed strongylid eggs. The FECRT conducted in these two groups revealed the IVM resistance in both (Table 2).

Next, the efficacy of the PYR was tested in the group of males with at least six individuals (Fig. 1 and Table 1). The interval between IVM and PYR treatments was around two months (56 days). Before PYR treatment, 5/6 animals were high shedders. The low shedder male exhibited a FEC of 165 EPG. Fourteen days post-treatment, 5/6 males continued to shed strongylid eggs. Resistance to PYR was discovered in the group of males (Table 1). This result is consistent with the fact that the PYR resistance has increasingly emerged between and within countries over the past couple of decades (Nielsen, 2022). In earlier French studies dating back five to ten years, confirmed resistance for PYR was found in up to 23% of the groups tested (Traversa et al., 2012; Geurden et al., 2013; Sallé et al., 2017). It is therefore, not surprising to observe PYR resistance in our recruited farm; this resistance is probably now more widespread within the country and deserves to be studied.

Finally, the efficacy of IVM was again tested in a group of females and males (n = 11; Fig. 1). Resistance to IVM in strongylids being uncommon, the confirmation of a reduced efficacy with several tests is strongly recommended by Kaplan et al. (2023) before declaring that IVM resistance is present in a farm. An interval of approximately six months was left between the PYR and the IVM treatments. This period minimizes the impact of the PYR treatment on the worm infrapopulation and ensure that it is representative of the overall worm population on the farm. Before treatment, 6/11 horses could be considered as high shedders. The minimum FEC in low shedders was 105 EPG. Fourteen days post-treatment, 10/11 of the horses continued to shed strongylid eggs. IVM resistance was also demonstrated in this group at this time of year (Table 1).

In Thoroughbreds, the intensive breeding practices, frequent AH treatments and frequent movements between countries for raising and training young horses, but also for racing, sales, stallion visiting and breeding (i.e., natural cover) expose this population to the risk of AH resistance emerging and spreading resistant worms. The resistance to ML (IVM and/or MOX) in strongylids has already been reported in Thoroughbred stud farms importing horses from other countries, such as Ireland and the UK (Nielsen et al., 2020; Abbas et al., 2021; Bull et al., 2023).

The Thoroughbred horseracing industry is an essential sector of Ireland and the UK economy. The two countries complement each other; Ireland is the European leader in Thoroughbred production in terms of births (producing almost half of all Thoroughbred horses in Europe), and the UK trains the third highest proportion of top-ranked flat Thoroughbreds behind only the USA and Australia (Teagasc, 2022; IFHA - International Federation for Horseracing Authorities, 2023). With this structuring of the Thoroughbred sector, European or international regulations should be implemented to impose a quarantine procedure for all equids coming from foreign countries to limit the spread of ML resistant strongylids. This quarantine procedure could include FECRT with targeted AH molecules whose effectiveness in the structure has been proven.

To conclude, this study provides the first evidence of triple resistance to all three licenced classes of AH, and more specifically to three of the four molecules available: FBZ, PYR and IVM. MOX is not currently used on yearlings on this farm, so it remains the last line of defence against strongylids. These results made the owner of the farm aware of the state of the situation, and since the beginning of 2024, he has wanted to continue with regular coproscopic analysis and, in partnership with his veterinarian, to start implementing a strategy of targeted selective treatment on high shedders. Further field studies involving more farms and horses are required to assess the prevalence of PYR and ML resistance in France. In parallel, the setting up of a European or worldwide network to monitor resistance, in particular, in this at-risk population, the galloping racehorses, could make it possible to inform owners of the risk of importing multi-resistant parasites and to offer them recommendations and to impose instructions on them.

## Authors’ contributions

AM supervised and conducted this study. AM and NL performed the faecal egg counts. AM and JCV carried out the data analysis. All the authors critically revised the article and approved the final version before submission.

## Funding sources

This work was supported by the 10.13039/501100007546French Agency for Food, Environmental and Occupational Health & Safety (Anses).

## Declaration of competing interest

The authors declare no conflicts of interest concerning the research, authorship, publication of this article and/or financial and personal relationships that could inappropriately influence this work.

## Conflicts of interest

The authors declare no conflicts of interest with respect to the research, authorship, publication of this article and/or financial and personal relationships that could inappropriately influence this work.
